# Prognostic biomarker GSTK1 in head and neck squamous cell carcinoma and its correlation with immune infiltration and DNA methylation

**DOI:** 10.3389/fgene.2023.1041042

**Published:** 2023-03-03

**Authors:** Yao Feng, Ying-Hui Zhou, Jie Zhao, Xiao-Lin Su, Ning-Xin Chen, Ya-Qiong Zhao, Qin Ye, Jing Hu, Ze-Yue Ou-Yang, Meng-Mei Zhong, Yi-Fan Yang, Peng-Ju Han, Yue Guo, Yun-Zhi Feng

**Affiliations:** ^1^ Department of Stomatology, The Second Xiangya Hospital, Central South University, Changsha, Hunan, China; ^2^ National Clinical Research Center for Metabolic Diseases, Hunan Provincial Key Laboratory of Metabolic Bone Diseases, and Department of Metabolism and Endocrinology, The Second Xiangya Hospital of Central South University, Changsha, Hunan, China; ^3^ Xiangya School of Stomatology, Central South University, Changsha, China; ^4^ College of Life Sciences, Sichuan University, Chengdu, Sichuan, China

**Keywords:** GSTK1, head and neck squamous cell carcinoma, prognosis, immune infiltration, DNA methylation

## Abstract

**Background:** Glutathione S-transferase kappa 1 (GSTK1) is critical in sarcoma and breast cancer (BRCA) development. However, the clinical significance of GSTK1 in head and neck squamous cell carcinoma (HNSC) remains unclear. This study is the first investigation into the role of GSTK1 in HNSC.

**Methods:** All original data were downloaded from the Cancer Genome Atlas (TCGA) dataset and verified by R Base Package 4.2.0. The expression of GSTK1 in various cancers was explored with TIMER and TCGA databases. Prognostic value of GSTK1 was analyzed *via* survival module of Kaplan-Meier plotter and Human Protein Atlas database and Cox regression analysis. The association between GSTK1 and clinical features was evaluated by Wilcoxon signed-rank test and logistic regression analysis. The relationship between GSTK1 and immune infiltration and methylation level was further explored. The expression of GSTK1 and its correlation with immune cell infiltration was verified by Immunohistochemical staining (IHC).

**Results:** GSTK1 was lower in HNSC, BRCA, Lung squamous cell carcinoma, and Thyroid carcinoma than in para-carcinoma. Low GSTK1 expression was associated with worse overall survival in Bladder urothelial carcinoma, Kidney renal papillary cell carcinoma, BRCA, and HNSC. However, only in BRCA and HNSC, GSTK1 expression in tumors was lower than that in normal tissues. Cox regression analyses confirmed that GSKT1 was an independent prognostic factor of overall survival in HNSC patients. The decrease in GSTK1 expression in HNSC was significantly correlated with high T stage and smoker history. IHC showed that the expression level of GSTK1 in HNSC was lower than that in para-carcinoma. In addition, GSEA showed that three pathways related to immune infiltration were positively correlated, while two pathways related to DNA methylation were negatively correlated with expression of GSTK1. Further analysis showed that GSTK1 was moderately positively correlated with the infiltration level of T cells and Cytotoxic cells, which was further confirmed by IHC. The methylation level of GSTK1 was associated with prognosis in patients with HNSC.

**Conclusion:** Low GSTK1 expression may be a potential molecular marker for poor prognosis in HNSC and provide new insight for the development of diagnostic marker or therapeutic target.

## Introduction

Head and neck squamous cell carcinoma (HNSC) is a highly heterogeneous malignant tumor that arises from the stratified mucosa of the upper airway and digestive tract, including the tongue, lips, tonsil, larynx, and pharynx (Peyrade et al., 2021). There are over 700,000 new HNSC cases per year, resulting in more than 450,000 annual deaths (Sung et al., 2021). HNSC ranks as the sixth most widespread and deadly cancer worldwide (Ferlay et al., 2010; Johnson et al., 2020). The 5-year survival rate for HNSC has not significantly increased over the past 30 years, and is only 30%–40% (Leemans et al., 2011; Seim et al., 2020). The main reason for this high mortality rate is that most patients with HNSC are diagnosed at an advanced stage (Hashim et al., 2019). The current standard of treatment for HNSC is surgical resection followed by chemotherapy and radiation (Johnson et al., 2020). However, once distant metastases are observed, HNSC cannot be cured in this way. Patients diagnosed with advanced stage HNSC have only a 34.9% survival rate (Chauhan et al., 2015). Identification of potential molecular diagnostic and therapeutic targets for HNSC remains crucial.

Glutathione S-transferases (GSTs) are a family of phase I enzymes that detoxify carcinogens to a variety of electrophilic compounds. GSTs are currently regarded as protective mechanisms against toxic substances and oxidative stress in the cellular adaptive response. Glutathione S-transferase kappa1 (GSTK1) is mitochondrial subfamily of GSTs that is localized to the mitochondria and known to catalyze the conjunction of glutathione into a wide range of hydrophobic substances, thereby actively protecting cellular macromolecules against oxidative stress. Altered levels of GSTK1 may influence the incidence and development of several cancers, including breast cancer, sarcoma, and prostate cancer. Higher expression of GSTK1 predicted the longer survival of patients with LumB breast cancer because GSTK1 is associated with decreased reactive species and oxidative stress (Luthra et al., 2018). Kun Quan et al. also showed that GSKT1 could predict the survival of patients with sarcomas based on gene expression and clinical data (Quan et al., 2022). A study using transgenic prostate adenocarcinoma in mice showed that low levels of GSTK1 were associated with hypermethylation, which affects tumorigenesis (Mavis et al., 2009). This information suggests that a comprehensive analysis of GSTK1 expression in cancer is necessary.

It has not yet been established that GSTK1 is a prognostic factor in HNSC, and its connection with HNSC has not been reported. The present work aimed to analyze the function of GSTK1 in the setting of HNSC. First, the expression of GSTK1 in human cancers was comprehensively analyzed. Next, the survival module of Kaplan-Meier Plotter database and Human Protein Atlas database (HPA) and Cox regression analysis were used to evaluate associations between GSTK1 expression and survival. Then, The Cancer Genome Atlas (TCGA) was utilized to analyze the clinical features of GSTK1 in HNSC. Immunohistochemical staining (IHC) was also used to verify the expression of GSTK1 and its correlation with immune cell infiltration in clinical specimens of HNSC patients. To gain a more in-depth understanding of the biological mechanisms underlying the effects of GSTK1, we performed gene set enrichment analysis (GSEA). Finally, we comprehensively explored the mechanisms between GSTK1 and tumorigenesis by analyzing immune infiltration and methylation in HNSC. This study suggests that GSTK1 may serve as a prognostic indicator and a therapeutic target for HNSC.

## Materials and methods

### Data and software availability

The TCGA database (http://cancergenome.nih.gov) is a landmark public cancer genomics program that has analyzed molecular characteristics of more than 20,000 primary cancer and normal samples that includes 33 cancer types, including adrenocortical carcinoma (ACC), bladder urothelial carcinoma (BLCA), breast invasive carcinoma (BRCA), cervical squamous cell carcinoma (CESC), cholangiocarcinoma (CHOL), colon adenocarcinoma (COAD), lymphoid neoplasm diffuse large B cell lymphoma (DLBC), esophageal carcinoma (ESCA), glioblastoma (GBM), brain lower grade glioma (LGG), head and neck squamous cell carcinoma (HNSC), kidney chromophobe (KICH), kidney renal clear cell carcinoma (KIRC), kidney renal papillary cell carcinoma (KIRP), acute myeloid leukemia (LAML), liver hepatocellular carcinoma (LIHC), lung adenocarcinoma (LUAD), lung squamous cell carcinoma (LUSC), mesothelioma (MESO), ovarian serous cystadenocarcinoma (OV), pancreatic adenocarcinoma (PAAD), pheochromocytoma and paraganglioma (PCPG), prostate adenocarcinoma (PRAD), rectum adenocarcinoma (READ), sarcoma (SARC), skin cutaneous melanoma (SKCM), stomach adenocarcinoma (STAD), testicular germ cell tumors (TGCT), thyroid carcinoma (THCA), thymoma (THYM), uterine corpus endometrial carcinoma (UCEC), uterine carcinosarcoma (UCS), and uveal melanoma (UVM).

The R Base Package 4.2.0 (R 4.2.0) was used to further evaluate this original data and verify the results obtained using the website database. Ethical approval for this study was obtained from the Medical Ethics Committee of the Second Xiangya Hospital of Central South University (KQ2019FY01). Detailed information on human cancers and corresponding individual sample sizes were collected from the Department of Stomatology at the Second Xiangya Hospital, Central South University (Supplementary Table S1). All applied online web tools were introduced below.

### GSTK1 expression in human cancers

The TIMER database (https://cistrome.shinyapps.io/timer/) was used to compare GSTK1 expression by human cancers and normal tissue (Li et al., 2017). Primary cancer and matched normal samples from the TCGA were also downloaded to evaluate GSTK1 expression.

### Association between GSTK1 expression and survival

We utilized the Kaplan-Meier plotter (kmplot.com/analysis) to assess the prognostic value of GSTK1 mRNA in various cancers based on overall survival (OS). Patient samples were divided into two cohorts around its median expression (high vs. low expression). Hazard ratios (HR) and the log-rank *p*-value of the 95% confidence interval (CI) were calculated on the webpage. A *p*-value < 0.05 was considered statistically significant. We used the HPA (https://www.proteinatlas.org) database to compare the relationships between the expression of GSTK1 mRNA and protein in various cancers and survival to verify the Kaplan-Meier plotter results. We also used the HPA database to obtain immunohistochemistry images of GSTK1 protein expression in normal versus cancerous tissues to explore its potential as a biomarker. In addition, the univariate and multivariate Cox regression analysis was used to evaluate the effect of GSTK1 expression level and other clinicopathological characteristics (M stage, N stage, T stage, Clinical stage, gender and age) on OS. Variables with *p* < 0.10 in univariate Cox regression analysis were further identified by multivariate Cox regression and the bilateral *p* < 0.05 was considered to be statistically significantly different.

### Association between GSTK1 expression and clinical characteristics

The RNAseq and clinical data of a total of 546 patients with HNSC were downloaded from the TCGA for further investigation. Unavailable or unknown clinical information was considered missing. A total of 502 RNAseq data with clinical information level 3 HTSeq-FPKM were for used for additional further analysis. A cut-off value for GSTK1 expression was defined as the median gene expression, and HNSC samples were divided into low- and high-expression groups. Relationships between GSTK1 expression and clinicopathological characteristics were assessed using wilcoxon signed-rank test and univariate and multivariate logistic regression analysis. Odds Ratio (OR) and the 95% CI were calculated on the R 4.2.0. For all statistical analyses, *p* < 0.05 was considered statistically significant. Our study was performed according to the publication guidelines provided by the TCGA.

### Gene set enrichment analysis (GSEA)

GSEA is a computational method that is based on the entire gene expression matrix (Subramanian et al., 2005). In this study, GSEA generated an ordered list of all genes according to their correlation with GSTK1 expression, and the number of permutations was set to 1,000. To be deemed statistically significant, the threshold value for statistical significance was set as *p* < 0.05 and FDR < 0.25 after correction. The adjusted *p*-value and normalized enrichment score (NES) were used to sort the first twenty positively pathways and the first twenty negatively pathways enriched in each phenotype. GSEA enrichment and visualization were analyzed using the ClusterProfiler version 3.11 package (Yu et al., 2012).

### GSTK1 expression and immune cell infiltration

The marker gene of 24 immune cells was extracted from the study by Bindea et al.(Bindea et al., 2013). Single-sample GSEA (ssGSEA) (Finotello and Trajanoski, 2018) was used to calculate the level of the microenvironment of the immune system based on GSTK1 mRNA TPM data. A Spearman correlation was used to correlate GSTK1 expression and these 24 kinds of cells. A correlation coefficient (r) of more than 0.3 and less than 0.5 was considered a moderate correlation. Furthermore, to analyze the influence of GSTK1 expression on tumor-infiltrating immune cells we classified 502 tumor samples into two groups. Figures were generated with Creat Elegant Data Visualisations Using the Grammar of Graphics (ggplot2 3.3.6) with *p* < 0.05. We also used the heat map to reveal the ratios of different tumor-infiltrating immune cell subpopulations with GSVA (Hänzelmann et al., 2013) which ranged from strong to weak correlations.

### Immunohistochemistry staining

Immunohistochemistry images of GSTK1 protein expression in HNSC cancer and para-carcinoma tissues were also obtained (*n* = 11 each). Each sample was blocked in 5% BSA for 30 min at room temperature, washed with ddH_2_O and PBS, and then probed with the GSTK1 primary antibody (ET7109-58, 1/50) for 30 min at room temperature. Detection was performed using an HRP conjugated compact polymer system. DAB was used as the chromogen. Tissues were counterstained with hematoxylin and mounted with DPX. Furthermore, immune cell classification in HNSC tissues was detected by IHC. We evaluated the relative infiltration level of T cells and Cytotoxic cells with appropriate antibodies (CD3, P07766, 1/250, Servicebio, Wuhan, China; CD8, P07766, 1/250, Servicebio, Wuhan, China) (Yin et al., 2021; Qu et al., 2022). The IHC staining results of GSTK1 were analyzed through staining intensity analysis using a semiquantitative integration method. The number of T cells and Cytotoxic cells was calculated by the percentage of per 100 cells in three non-overlapping high-power fields (HPFs; ×100; 0.32 mm^2^), and the relative mean value was used for subsequent analysis. The evaluation of specimens was analyzed by two investigators independently who were blinded to the clinical information.

### Correlation between GSTK1 mutation and methylation

cBioPortal (www.cbioportal.org) was used to explore, analyze, and visualize poly-dimensional cancer genomics data (Gao et al., 2013). GSTK1 mRNA expression levels, mutation types, copy number alterations and methylation levels across the TCGA database were obtained using the “Cancer Types Summary” module of cBioPortal. MethSurv (https://biit.cs.ut.ee/methsurv/) was further used to correlate GSTK1 expression and methylation levels in TCGA-HNSC. MethSurv is a web tool for univariate and multivariate survival analyses based on DNA methylation biomarkers, including 25 different types of cancer and 7,358 patients (Modhukur et al., 2018). The present work compared the genetic methylation of GSTK1 mRNA and its association with OS.

## Results

### Significant expression of GSTK1 between tumor and normal tissues in human cancer

**FIGURE 1:**
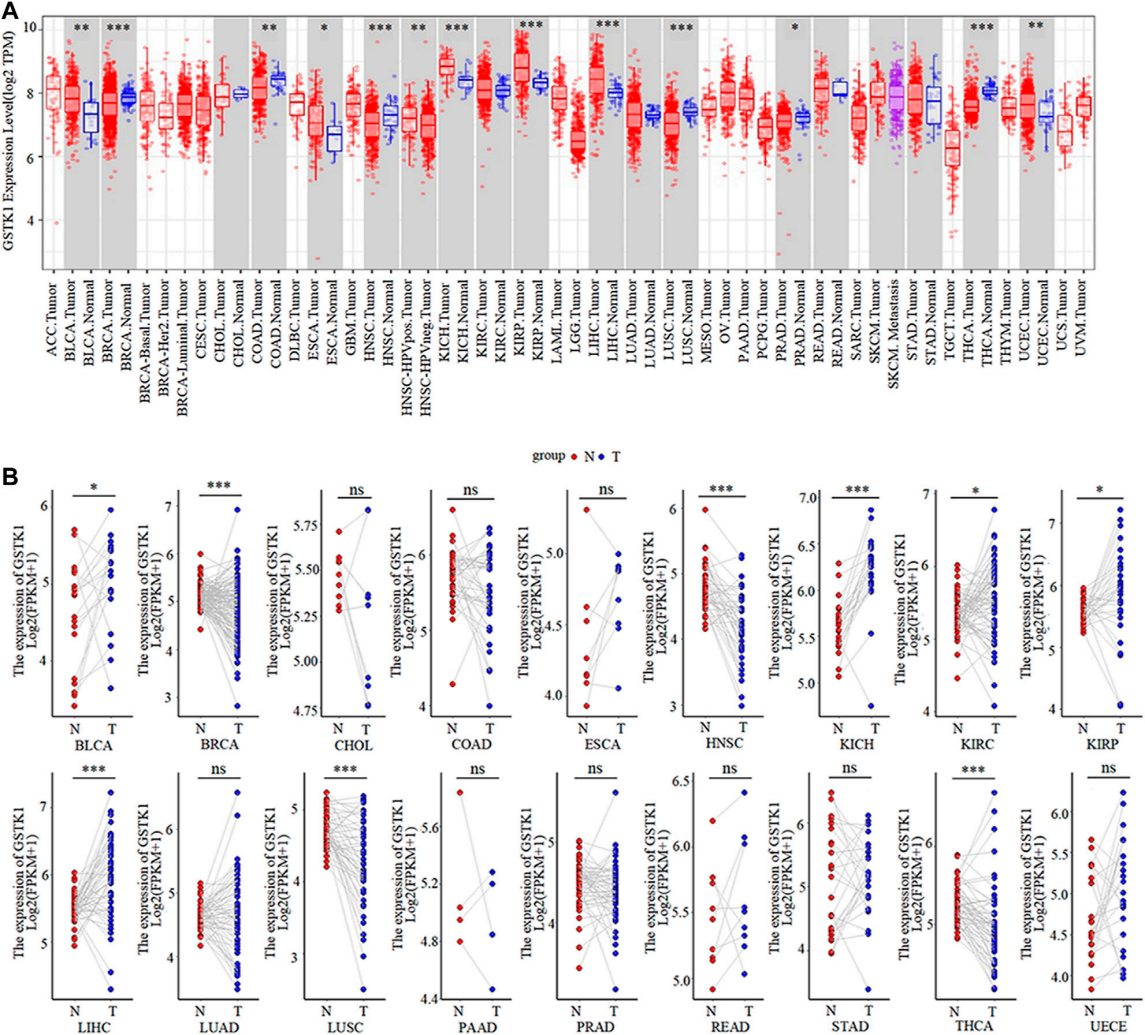
GSTK1 expression in various types of human cancers **(A)** Comparison of the GSTK1 expression between various cancers and normal tissue using the TIMER database. **(B)** GSTK1 expression of several cancers vs. paired normal tissue from the TCGA database. (*p < 0.05, **p < 0.01, ***p < 0.001). (ACC: adrenocortical carcinoma; BLCA: bladder urothelial carcinoma; BRCA, breast invasive carcinoma; CESC, cervical squamous cell carcinoma; CHOL: cholangiocarcinoma; COAD: colon adenocarcinoma; DLBC, lymphoid neoplasm diffuse large B cell lymphoma; ESCA, esophageal carcinoma; GBM, glioblastoma; LGG, brain lower grade glioma; HNSC, head and neck squamous cell carcinoma; KICH, kidney chromophobe; KIRC, kidney renal clear cell carcinoma; KIRP, kidney renal papillary cell carcinoma; LAML, acute myeloid leukemia; LIHC, liver hepatocellular carcinoma; LUAD, lung adenocarcinoma; LUSC, lung squamous cell carcinoma; MESO, mesothelioma; OV, ovarian serous cystadenocarcinoma; PAAD, pancreatic adenocarcinoma; PCPG, pheochromocytoma and paraganglioma; PRAD, prostate adenocarcinoma; READ, rectum adenocarcinoma; SARC, sarcoma; SKCM, skin cutaneous melanoma; STAD, stomach adenocarcinoma; TGCT, testicular germ cell tumors; THCA, thyroid carcinoma; THYM, thymoma; UCEC, uterine corpus endometrial carcinoma; UCS, uterine carcinosarcoma; UVM, uveal melanoma).

Based on our analysis of the TIMER database, GSTK1 had inconsistent mRNA expression in the 34 types of human common cancer. Compared with that in normal tissues, GSTK1 expression was significantly higher in BLCA, ESCA, KICH, KIRP, LIHC, and UCEC. However, it was lower in BRCA, COAD, HNSC, LUSC, PRAD and THCA (Figure 1A). The results from the TCGA database analysis are shown as complementary results for cancers without paired normal tissues in the TIMER database (Figure 1B). GSTK1 mRNA expression was also significantly higher in BLCA, KICH, KIRP and LIHC, which was consistent with the TIMER database results. Decreased GSTK1 mRNA expression compared with normal tissues was consistently observed in HNSC, BRCA, LUSC, and THCA. The above results suggested that the GSTK1 expression level may be related to tumorigenesis.

### Decreased expression of GSTK1 correlating with poor outcome

**FIGURE 2:**
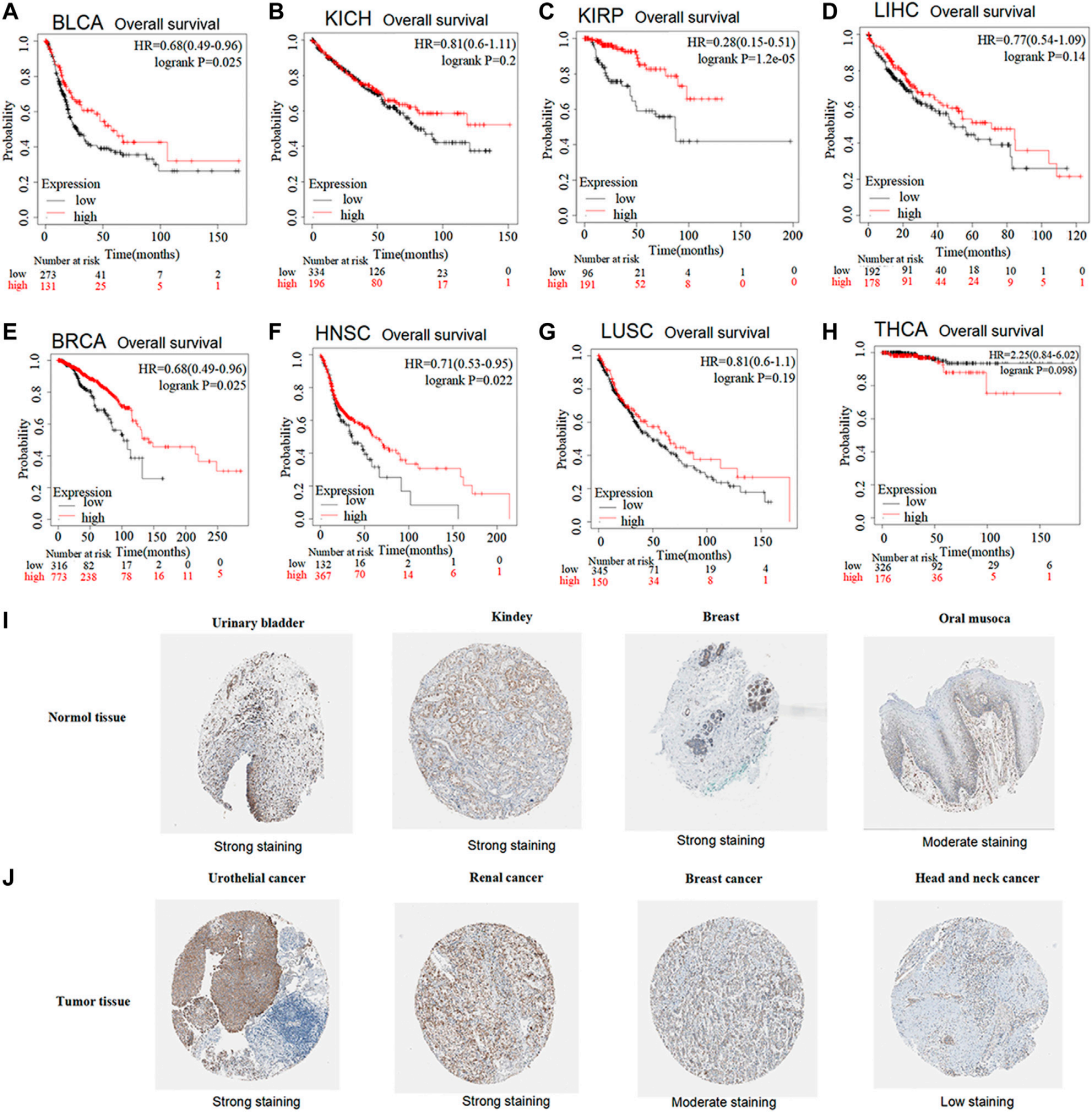
Prognostic value of low vs. high GSTK1 expression analyzed with the Kaplan-Meier plotter database and the Human Protein Atlas (HPA) database **(A–H)** Kaplan–Meier survival curves for BLCA **(A)**, KICH **(B)**, KIRP **(C)**, LIHC **(D)**, BRCA **(E)**, HNSC **(F)**, LUSC **(G)**, THCA **(H)** tumors with high and low GSKT1 expression analyzed using the Kaplan-Meier plotter database **(I)** Validation of the expression level of GSTK1 in various normal tissues using the HPA database (immunohistochemistry). **(J)** Validation of the expression level of GSTK1 in various tumors using the HPA database (immunohistochemistry). (BLCA, bladder urothelial carcinoma; KICH, kidney chromophobe; LIHC, liver hepatocellular carcinoma; BRCA, breast invasive carcinoma; HNSC, head and neck squamous cell carcinoma; LUSC, lung squamous cell carcinoma; THCA, thyroid carcinoma).

The prognostic value of GSTK1 expression in human cancers was analyzed using the Kaplan-Meier plotter database and the HPA database. In the Kaplan-Meier plotter database, we found that lower GSTK1 expression was associated with worse OS in BLCA (HR = 0.68, *p* < 0.01, Figure 2A), KIRP (HR = 0.28, *p* < 0.001 Figure 2C), BRCA (HR = 0.68, *p* < 0.05, Figure 2E), and HNSC (HR = 0.71, *p* < 0.05 Figure 2F). However, there was no significant correlation with prognosis in KICH, LIHC, LUSC, and THCA. At the same time, we analyzed the relationship between GSTK1 expression and 5-year survival using the HPA database as shown in Table 1. Lower expression of GSTK1 was associated with worse 5-year survival in BLCA (50% vs. 37%, *p* < 0.05), KIRP (83% vs. 58%, *p* < 0.001), BRCA (86% vs. 65%, *p* < 0.001), and HNSC (50% vs. 27%, *p* < 0.001). Immunohistochemical results on GSTKI protein expression based on the HPA database are shown in Figures 2I, J. The expression of GSTK1 protein in BLCA and KIRP in tumor tissues was the same as in adjacent tissues, which was contrary to the results obtained from the TIMER database. However, the expression level of GSTK1 protein in BRCA and HNSC tumors was lower than that of normal tissues, which was consistent with TIMER database findings. These data verify that GSTK1 is less expressed in BRCA and HNSC. Univariate and multivariate Cox regression analyses of clinical variables were further performed to identify whether GSTK1 was an independent prognostic predictor of OS in HNSC patients. In univariate Cox regression analysis, GSTK1 expression level (HR = 0.806, 95% CI = 0.636–1.002, *p* < 0.10), M stage (HR = 4.675, 95% CI = 1.722–12.691, *p* < 0.01), gender (HR = 1.333, 95% CI = 1.002–1.773, *p* < 0.05) and age (HR = 1.020, 95% CI = 1.008–1.003, *p* < 0.01) were associated with OS. Further analysis by multivariate Cox regression showed that GSKT1 expression (HR = 0.772, 95% CI = 0.605–0.984, *p* < 0.05), M stage (HR = 5.591, 95% CI = 2.038–15.334, *p* < 0.001), and age (HR = 1.021, 95% CI = 1.007–1.032, *p* < 0.01) were independent prognostic factors of OS (Table 2). These results indicated that low GSK1 expression was associated with poor prognosis of HNSC patients.

**TABLE 1:** Survival analysis of GSTK1 between BLCA, KIRP, BRCA, and HNSC using The Human Protein Atlas.

	BLCA	KIRP	BRCA	HNSC
Best expression cut off (FPKM)	44.31	58.42	21.14	13.91
Median expression (FPKM)	36.63	70.76	29.3	19.65
Median follow up time (Years)	1.44	2.11	2.37	1.75
*p-*value	0.021	0.000	0.000	0.011
5- year survival high expression	50%	83%	86%	50%
5-year survival low expression	37%	58%	65%	27%

**TABLE 2:** Univariate and multivariate COX regression analyses of GSTK1 expression level and clinical factors on prognosis.

Characteristics	Univariate analysis				Multivariate analysis		
	Hazard ratio	95% CI	*p*-value		Hazard ratio	95% CI	*p*-value
GSTK1 expression	0.806	0.636–1.022	0.076		0.772	0.605–0.984	**0.036**
M stage	4.675	1.722–12.691	**0.002**		5.591	2.038–15.334	**0.000**
N stage	1.222	0.936–1.595	0.141				
T stage	1.225	0.941–1.674	0.123				
Clinical stage	1.200	0.866–1.662	0.274				
Gender	1.333	1.002–1.773	**0.048**		1.213	0.897–1.640	0.211
Age	1.020	1.008–1.033	**0.002**		1.021	1.007–1.034	**0.002**

Notes: Bold values mean p-value is significant (*p* < 0.05).

### Associations between GSTK1 expression and HNSC clinical characteristics

**FIGURE 3:**
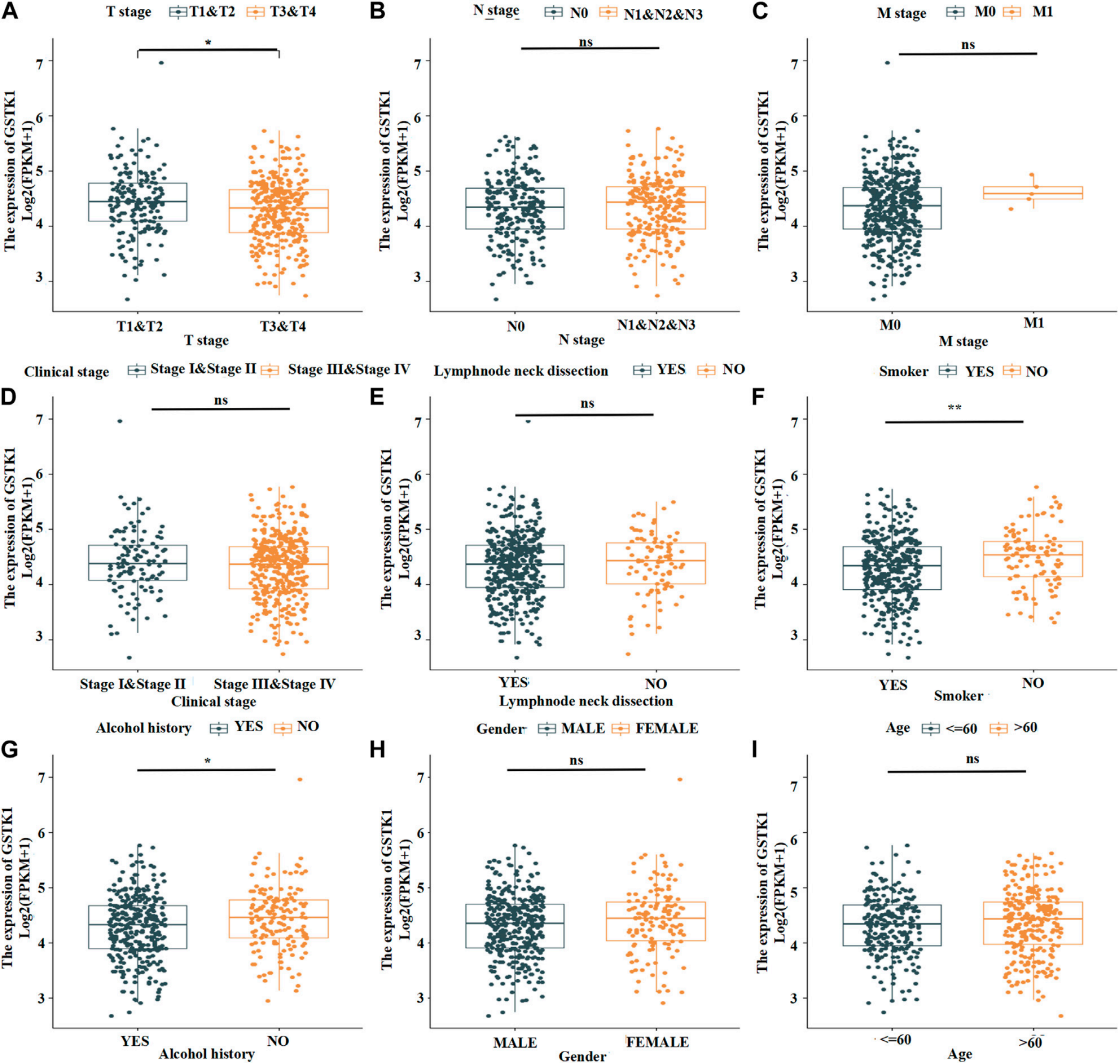
Expression patterns of GSTK1 mRNA in the tumors of patients with different clinical characteristics in TCGA. **(A)** The most significant difference was from T stage 1-2 to T stage 3-4. **(B)** Expression was not significantly different from N stage 0 to N stage 1–3. **(C)** The mRNA was not significantly different from M stage 0 to M stage 1. **(D)** Expression in Clinical stage III-IV was lower than stage I-II but not significantly different. **(E)** Expression in HNSC tumors following a lymph node dissection was lower than those who did not undergo a lymph node dissection but not significantly different. **(F, G)** mRNA expression was much lower in HNSC patients with a smoking and significant alcohol history. **(H, I)** mRNA expression did not differ between gender and age. (*p < 0.05, **p < 0.01, ***p < 0.001).

We analyzed the mRNA expression levels of GSTK1 in different clinical categories of the TCGA database. Associations identified between GSTK1 expression and clinical features is summarized in Table 3. Similar results are shown in Figure 3. There was a significant correlation between low GSTK1 expression and higher T stage (*p* < 0.001, Figure 3A), active smoking (*p* < 0.01, Figure 3F), and a history of significant alcohol intake (*p* < 0.05, Figure 3G). Univariate logistic regression of GSTK1 expression (Table 4) revealed that GSTK1 expression was associated with T stage (T3&T4 vs. T1&T2, OR = 0.664, 95%CI: 0.443–0.934, *p* < 0.05), active smoking (Yes vs. No, OR = 0.617, 95% CI: 0.400–0.945, *p* < 0.05), and a history of significant alcohol intake (No vs. Yes, OR = 1.535, 95% CI: 1.151–1.919, *p* < 0.05). Further analysis by multivariate logistic regression showed that T stage (T3&T4 vs. T1&T2, OR = 0.671, 95%CI: 0.285–1.058, *p* < 0.05) and smoker history (Yes vs. No, OR = 0.620, 95% CI: 0.233–1.008, *p* < 0.05) were independent significant factors of GSTK1 expression (all *p* < 0.05, Table 4).

**TABLE 3:** Associations between GSTK1 expression and the clinicopathological features of HNSC.

Characteristic	Low expression of GSTK1	High expression of GSTK1	*p-*value
n	251	251	
T stage, n (%)			0.082
T1	13 (2.7%)	20 (4.1%)	
T2	63 (12.9%)	81 (16.6%)	
T3	66 (13.6%)	65 (13.3%)	
T4	101 (20.7%)	78 (16%)	
N stage, n (%)			0.198
N0	124 (25.8%)	115 (24%)	
N1	43 (9%)	37 (7.7%)	
N2	67 (14%)	87 (18.1%)	
N3	5 (1%)	2 (0.4%)	
M stage, n (%)			0.214
M0	239 (50.1%)	233 (48.8%)	
M1	1 (0.2%)	4 (0.8%)	
Clinical stage, n (%)			0.897
Stage I	10 (2%)	9 (1.8%)	
Stage II	45 (9.2%)	50 (10.2%)	
Stage III	49 (10%)	53 (10.9%)	
Stage IV	139 (28.5%)	133 (27.3%)	
Lymph node dissection, n (%)			0.403
No	41 (8.2%)	49 (9.8%)	
Yes	209 (41.9%)	200 (40.1%)	
Smoker, n (%)			0.035
No	45 (9.1%)	66 (13.4%)	
Yes	200 (40.7%)	181 (36.8%)	
Alcohol history, n (%)			0.046
No	68 (13.8%)	90 (18.3%)	
Yes	177 (36%)	156 (31.8%)	
Gender, n (%)			0.267
Female	61 (12.2%)	73 (14.5%)	
Male	190 (37.8%)	178 (35.5%)	
Age, median (SD)	60.11 (11.66)	62.05 (12.08)	0.068

**TABLE 4:** Logistic analysis of associations between GSTK1 expression and clinical characteristics.

Characteristics	Total(N)	Univariate analysis		Multivariate analysis	
		Odds ratio (95% CI)	*p*-value	Odds ratio (95% CI)	*p-*value
T stage (T3&T4 vs. T1&T2)	487	0.664 (0.443–0.934)	**0.021**	0.671 (0.285–1.058)	**0.043**
N stage (N1&N2&N3 vs. N0)	480	1.198 (0.847–1.550)	0.313		
M stage (M1 vs. M0)	477	3.644 (1.446–5.841)	0.249		
Clinical_stage (Stage III&Stage IV vs. Stage I&Stage II)	488	0.930 (0.511–1.349)	0.734		
Lymphnode neck dissection (Yes vs. No)	499	1.203 (0.743–1.663)	0.430		
Smoker (Yes vs. No)	492	0.617 (0.400–0.945)	**0.027**	0.620 (0.233–1.008)	**0.016**
Alcohol history (No vs. Yes)	491	1.535 (1.151–1.919)	**0.029**	1.434 (1.038–1.829)	0.074
Gender (Female vs. Male)	502	1.178 (0.780–1.576)	0.419		
Age (>60 vs. ≤60)	501	0.836 (0.485–1.187)	0.317		

Notes: Bold values mean *p*-value is significant (*p* < 0.05).

### Validation of GSKT1 expression in HNSC using clinical specimens

**FIGURE 4:**
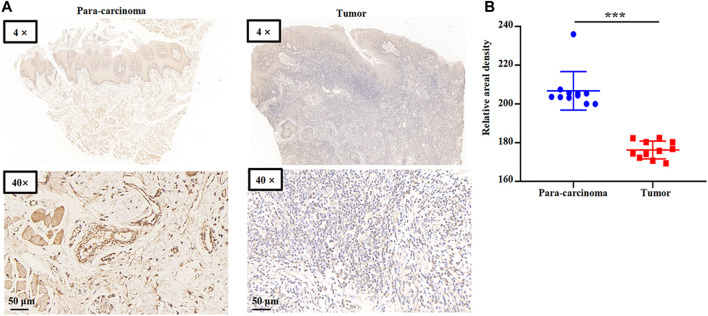
Representative immunohistochemical staining for GSTK1 protein in HNSC **(A)** Immunohistochemical staining of GSTK1 was performed in tumor (*n* = 11) and para-carcinoma tissues (*n* = 11). Representative images are shown. Score bars, 50 μm. **(B)** Staining was quantified as shown. The dot plot depicts the mean and standard deviation of 11 images of tumor and adjacent normal tissues. (***p < 0.001).

We collected HNSC clinical specimens to measure GSTK1 expression. GSTK1 expression in the tumors was lower than that of para carcinomatous tissues (Figures 4A, B). These data verify that GSTK1 is under-expressed in HNSC.

### GSTK1 signaling pathways identified using GSEA

**FIGURE 5:**
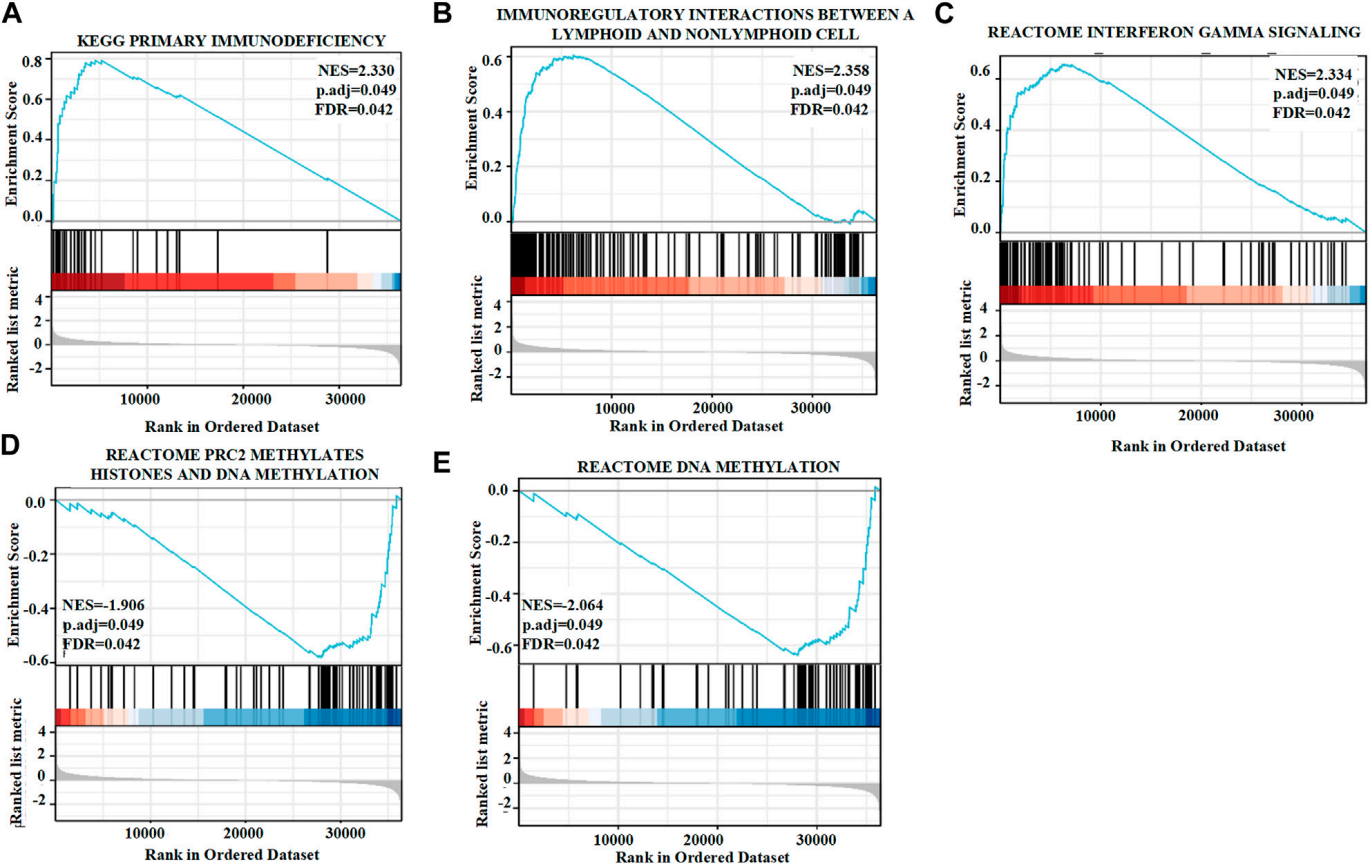
Enrichment plots from gene set enrichment analysis (GSEA). GSTK1 was differentially enriched in the setting of a primary immunodeficiency **(A)**, immunoregulatory interactions between lymphoid and a non-lymphoid cells **(B)**, interferon gamma signaling **(C)**, PRC2 methylates histones **(D)**, and DNA methylation **(E)**. NES: normalized enrichment score; FDR: false discovery rate.

Approximately 20,000 differentially expressed genes were identified between the high and low GSEA expression groups created using the TCGA. Based on the normalized enrichment score (NES), we selected the first twenty positive or negative enrichment signaling pathways with high or low GSTK1 gene expression (Figure 5; Tables 5, 6). As shown in Figure 5, GSTK1-related HNSC was associated with immune infiltration and DNA methylation. Three pathways related to immune infiltration were positively correlated, including primary immunodeficiency (Figure 5A), Immunoregulatory interactions between a lymphoid and a non-lymphoid cell (Figure 5B), and Interferon gamma signaling (Figure 5C). Two pathways related to DNA methylation were negatively correlated with expression of GSTK1, including PRC2 methylates histones (Figure 5D), and DNA methylation (Figure 5E).

**TABLE 5:** The First Twenty positively correlated groups of gene set enrichment analysis (GSEA) based on their normalized enrichment score (NES) and *p*-value in HNSC.

Description	Set size	Enrichment score	NES	*p-*value	*p*.adjust	*q* values	Rank	Leading_edge
REACTOME_GENERATION_OF_SECOND_MESSENGER_MOLECULES	37	0.850	2.541	0.002	0.048	0.042	3,547	tags = 78%, list = 10%, signal = 71%
KEGG_INTESTINAL_IMMUNE_NETWORK_FOR_IGA_PRODUCTION	46	0.816	2.532	0.002	0.048	0.042	4,098	tags = 72%, list = 11%, signal = 64%
KEGG_ANTIGEN_PROCESSING_AND_PRESENTATION	81	0.729	2.504	0.002	0.048	0.042	2,394	tags = 42%, list = 7%, signal = 39%
WP_ALLOGRAFT_REJECTION	89	0.716	2.501	0.002	0.048	0.042	4,191	tags = 58%, list = 11%, signal = 52%
REACTOME_PD_1_SIGNALING	26	0.897	2.484	0.002	0.048	0.042	2,260	tags = 88%, list = 6%, signal = 83%
KEGG_ALLOGRAFT_REJECTION	35	0.836	2.479	0.002	0.048	0.042	4,000	tags = 80%, list = 11%, signal = 71%
KEGG_GRAFT_VERSUS_HoT_DISEASE	37	0.818	2.445	0.002	0.048	0.042	2,697	tags = 73%, list = 7%, signal = 68%
REACTOME_COSTIMULATION_BY_THE_CD28_FAMILY	72	0.720	2.428	0.002	0.048	0.042	2,697	tags = 46%, list = 7%, signal = 43%
**KEGG_PRIMARY_IMMUNODEFICIENCY**	35	0.817	2.421	0.002	0.048	0.042	4,939	tags = 69%, list = 13%, signal = 59%
PID_IL12_2PATHWAY	62	0.730	2.395	0.002	0.048	0.042	3,118	tags = 50%, list = 9%, signal = 46%
KEGG_AUTOIMMUNE_THYROID_DISEASE	50	0.755	2.369	0.002	0.048	0.042	4,000	tags = 58%, list = 11%, signal = 52%
KEGG_TYPE_I_DIABETES_MELLITUS	41	0.769	2.346	0.002	0.048	0.042	2,704	tags = 66%, list = 7%, signal = 61%
WP_SELECTIVE_EXPRESSION_OF_CHEMOKINE_RECEPTORS_DURING_TCELL_POLARIZATION	29	0.804	2.301	0.002	0.048	0.042	3,783	tags = 62%, list = 10%, signal = 56%
PID_IL12_STAT4_PATHWAY	32	0.784	2.283	0.002	0.048	0.042	4,235	tags = 59%, list = 12%, signal = 53%
BIOCARTA_TH1TH2_PATHWAY	21	0.870	2.282	0.002	0.048	0.042	3,979	tags = 81%, list = 11%, signal = 72%
KEGG_ASTHMA	28	0.799	2.273	0.002	0.048	0.042	3,668	tags = 68%, list = 10%, signal = 61%
REACTOME_TCR_SIGNALING	122	0.628	2.269	0.002	0.048	0.042	2,260	tags = 27%, list = 6%, signal = 25%
**REACTOME_IMMUNOREGULATORY_INTERACTIONS_BETWEEN_A_LYMPHOID_AND_A_NON_LYMPHOID_CELL**	186	0.592	2.260	0.002	0.048	0.042	6,416	tags = 51%, list = 18%, signal = 42%
**REACTOME_INTERFERON_GAMMA_SIGNALING**	91	0.637	2.231	0.002	0.048	0.042	6,928	tags = 56%, list = 19%, signal = 46%
KEGG_HEMATOPOIETIC_CELL_LINEAGE	84	0.626	2.160	0.002	0.048	0.042	3,614	tags = 43%, list = 10%, signal = 39%

Notes: Bold values mean pathways related to immune infiltration.

**TABLE 6:** The First Twenty negatively correlated groups of gene set enrichment analysis (GSEA) in HNSC.

Description	Set Size	Enrichment score	NES	*p-*value	p.adjust	*q*values	Rank	Leading_edge
REACTOME_FORMATION_OF_THE_CORNIFIED_ENVELOPE	128	−0.756	−2.816	0.002	0.048	0.042	3238	Tags = 66%, list = 9%, signal = 61%
REACTOME_KERATINIZATION	216	−0.663	−2.647	0.002	0.048	0.042	2531	Tags = 43%, list = 7%, signal = 40%
REACTOME_RUNX1_REGULATES_GENES_INVOLVED_IN_MEGAKARYOCYTE_DIFFERENTIATION_AND_PLATELET_FUNCTION	96	−0.597	−2.132	0.002	0.048	0.042	8691	Tags = 57%, list = 24%, signal = 44%
REACTOME_TRANSCRIPTIONAL_REGULATION_BY_SMALL_RNAS	105	−0.580	−2.112	0.002	0.048	0.042	9937	Tags = 59%, list = 27%, signal = 43%
REACTOME_ERCC6_CSB_AND_EHMT2_G9A_POSITIVELY_REGULATE_RRNA_EXPRESSION	74	−0.616	−2.103	0.002	0.048	0.042	10021	Tags = 68%, list = 27%, signal = 49%
REACTOME_HDACS_DEACETYLATE_HISTONES	91	−0.593	−2.100	0.002	0.048	0.042	8865	Tags = 59%, list = 24%, signal = 45%
REACTOME_HCMV_LATE_EVENTS	113	−0.572	−2.093	0.002	0.048	0.042	9937	Tags = 55%, list = 27%, signal = 40%
REACTOME_HCMV_EARLY_EVENTS	135	−0.557	−2.089	0.002	0.048	0.042	7226	Tags = 45%, list = 20%, signal = 36%
REACTOME_ACTIVATED_PKN1_STIMULATES_TRANSCRIPTION_OF_AR_ANDROGEN_RECEPTOR_REGULATED_GENES_KLK2_AND_KLK3	65	−0.633	−2.080	0.002	0.048	0.042	7226	Tags = 57%, list = 20%, signal = 46%
REACTOME_RHO_GTPASES_ACTIVATE_PKNS	92	−0.583	−2.075	0.002	0.048	0.042	8717	Tags = 58%, list = 24%, signal = 44%
REACTOME_B_WICH_COMPLEX_POSITIVELY_REGULATES_RRNA_EXPRESSION	89	−0.589	−2.075	0.002	0.048	0.042	7226	Tags = 45%, list = 20%, signal = 36%
REACTOME_SIRT1_NEGATIVELY_REGULATES_RRNA_EXPRESSION	66	−0.629	−2.074	0.002	0.048	0.042	7226	Tags = 53%, list = 20%, signal = 43%
**REACTOME_DNA_METHYLATION**	63	−0.630	−2.065	0.002	0.048	0.042	7226	Tags = 56%, list = 20%, signal = 45%
REACTOME_POSITIVE_EPIGENETIC_REGULATION_OF_RRNA_EXPRESSION	104	−0.563	−2.047	0.002	0.048	0.042	10021	Tags = 56%, list = 27%, signal = 41%
WP_NRF2_PATHWAY	145	−0.538	−2.036	0.002	0.048	0.042	4991	Tags = 50%, list = 14%, signal = 44%
REACTOME_CONDENSATION_OF_PROPHASE_CHROMOSOMES	72	−0.598	−2.030	0.002	0.048	0.042	8061	Tags = 53%, list = 22%, signal = 41%
**REACTOME_PRC2_METHYLATES_HISTONES_AND_DNA**	71	−0.596	−2.012	0.002	0.048	0.042	4406	Tags = 52%, list = 12%, signal = 46%
REACTOME_REGULATION_OF_CHOLESTEROL_BIOSYNTHESIS_BY_SREBP_SREBF_	55	−0.626	−2.000	0.002	0.048	0.042	7876	Tags = 64%, list = 22%, signal = 50%
REACTOME_HCMV_INFECTION	159	−0.525	−2.000	0.002	0.048	0.042	9067	Tags = 48%, list = 25%, signal = 37%
REACTOME_OXIDATIVE_STRESS_INDUCED_SENESCENCE	122	−0.488	−1.729	0.002	0.0048	0.042	8451	Tags = 49%, list = 23%, signal = 38%

Notes: Bold values mean pathways related to DNA methylation

### GSTK1 gene expression correlates with immune infiltration in HNSC

**FIGURE 6:**
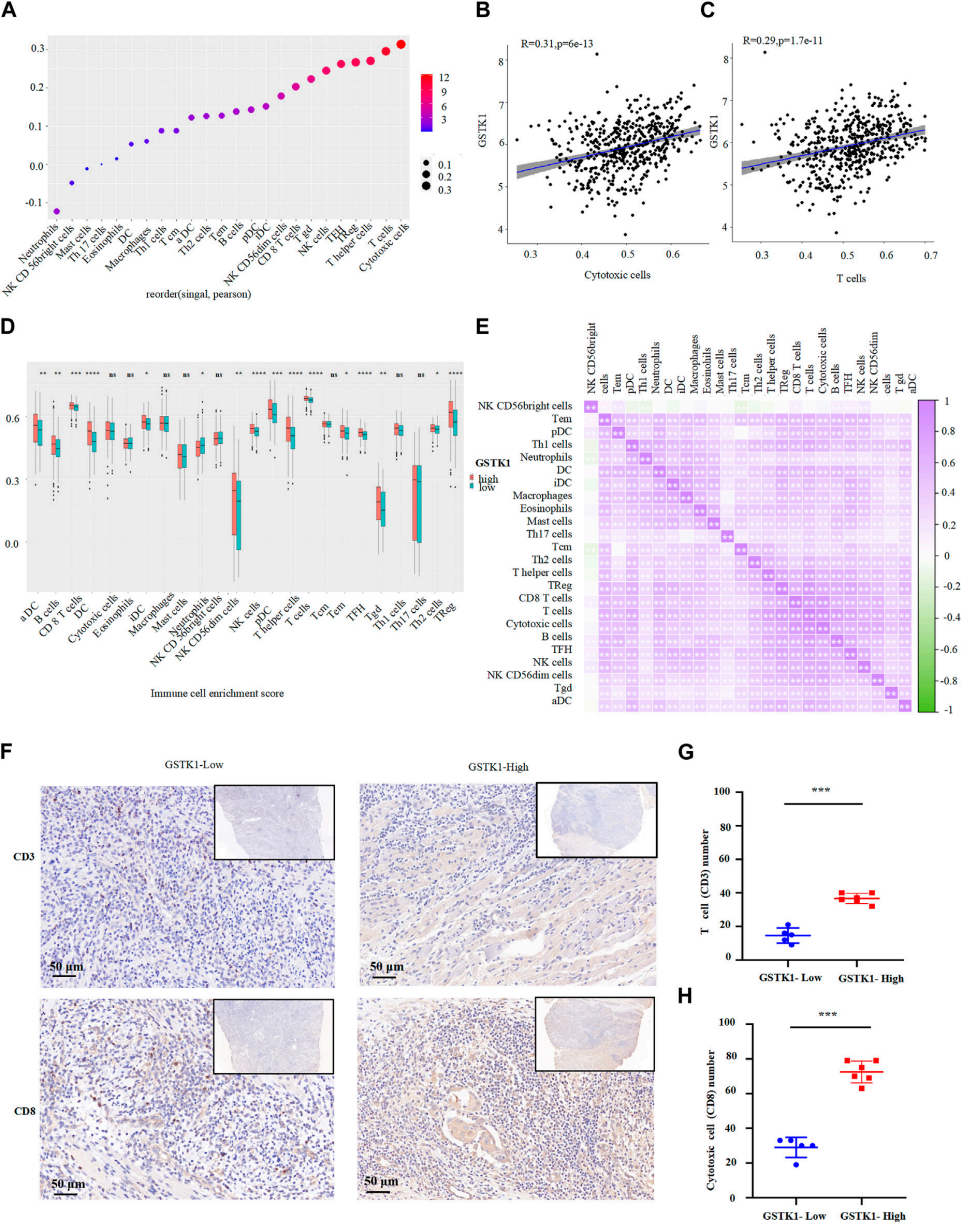
Association of GSTK1 gene expression with immune infiltration. **(A)** Association analysis between GSTK1 expression and immune cells. **(B, C)** Association analysis between GSTK1 expression and the immune infiltration levels of T cells and Cytotoxic cells. **(D)** The varied proportions of 24 subtypes of immune cells between low and high GSTK1 expression groups in HNSC samples. **(E)** Heatmap of 24 immune infiltration cells in tumor samples. **(F)** Immunohistochemical staining of CD3 and CD8 were performed in tumor tissues from GSTK1-high (*n* = 6) and GSTK1-low patients groups (*n* = 5). Score bars, 50 μm. **(G)** Comparison of T cell (CD3) counts in GSTK1-high and GSTK1-low tumor tissues from HNSC patients. **(H)** Comparison of Cytotoxic cells (CD8) counts in GSTK1-high and GSTK1-low tumor tissues from HNSC patients. (*p < 0.05, **p < 0.01, ***p < 0.001).

The above results indicate that GSTK1 is involved in immune cell infiltration and the inflammatory response, which are independent predictors of cancer survival (Figure 5A–C). We therefore investigated the relationship between GSTK1 expression and immune cells in HNSC. We evaluated correlations between GSTK1 and 24 immune cell subtypes in HNSC (Figure 6A), and found that GSTK1 has a close positive correlation with Cytotoxic cells, T cells, NK CD56dim cells, TReg, aDC, TFH, T helper cells, and CD8 T cells (Figure 6A). Further analysis showed that GSTK1 expression was moderately positively associated with the infiltration of Cytotoxic cells (*R* = 0.31, *p* < 0.001, Figure 6B) and T cells (*R* = 0.29, *p* < 0.001, Figure 6C). We also tried to determine whether the tumor immune micro-environment was different in HNSC patients with low GSTK1 levels compared with those with high expression levels. In total, 502 HNSC samples were divided into two groups based on GSTK1 expression, with 251 samples in the low expression group and 251 samples in the high expression group (Figure 6D). aDC, B cells, iDC, Neutrophils, NK CD56dim cells, Tem, Tgd and Th2 cells were affected by GSTK1 expression, with considerable differences observed in CD8 T cells, Cytotoxic cells, NK cells, pDC, T cells, T helper cells, TFH, and TReg between the low and high GSTK1 groups. We also evaluated possible correlations between 24 types of immune cells (Figure 6E). The immune cell co-expression correlation analysis revealed that most of the immune cells in the network had a strong positive correlation with each other, except for NK CD56 bright cells and Th17 cells. In order to verify GSTK1 expression correlation with Cytotoxic cells (CD8) and T cells (CD3) infiltration, we detected GSTK1, CD8 and CD3 expression in tumor samples. We divided the tumor samples into two groups according to GSTK1 expression and found that Cytotoxic cells and T cells infiltration were positively correlated with the level of GSTK1 (*p* < 0.001, Figures 6F–H). These data suggest that GSTK1 may promote the tumor immune response against HNSC by directing Cytotoxic cells and T cells infiltration into the tumors.

### Correlation between GSTK1 expression and methylation

**FIGURE 7:**
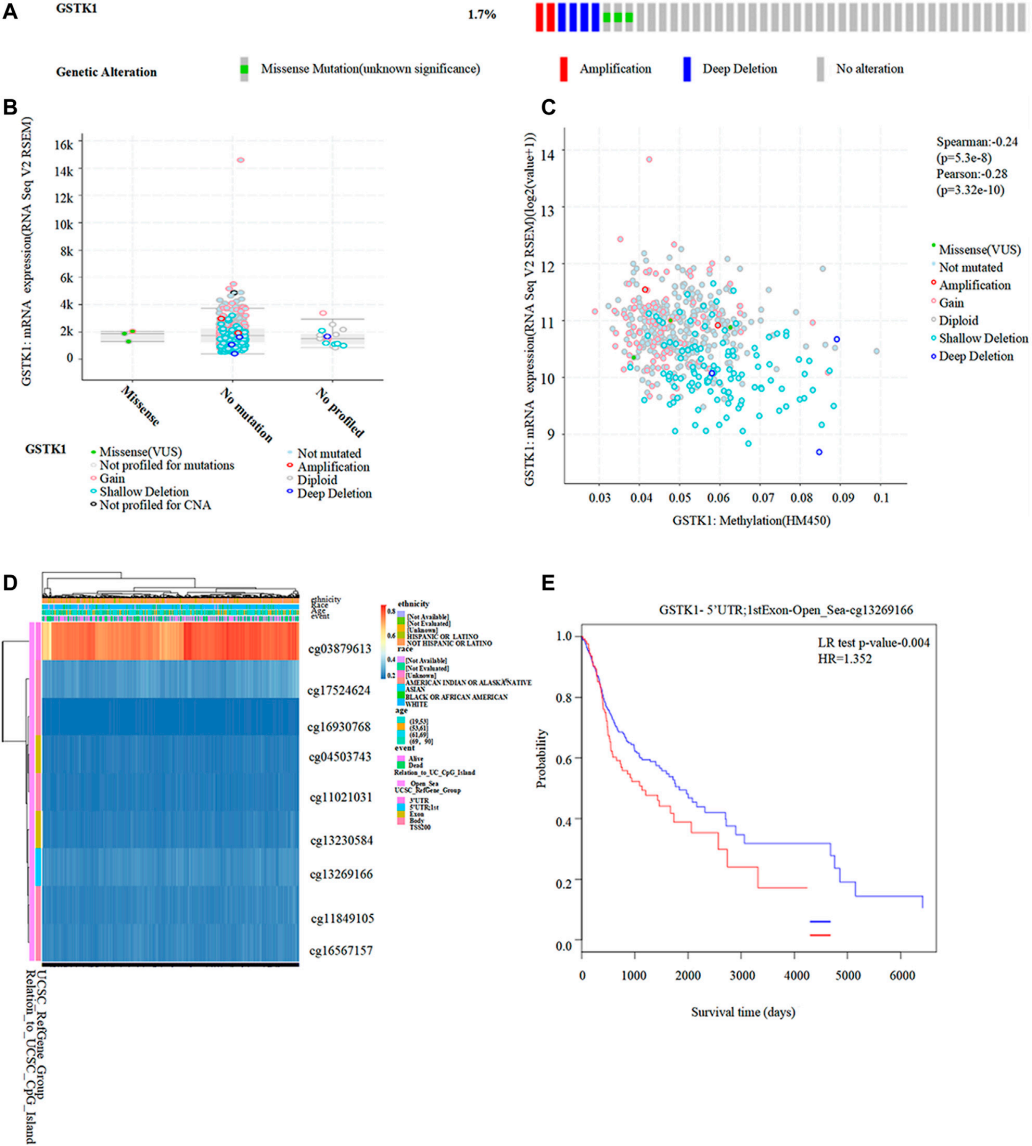
Correlation between GSTK1 expression and methylation in HNSC. **(A, B)** Genetic alterations in GSTK1 and their association with GSTK1 expression in HNSC patients using cBioportal. **(C)** Methylation levels of GSTK1 significantly correlated GSTK1 expression using cBioportal. **(D)** Visualization comparing methylation level and the GSTK1 expression. **(E)** Kaplan-Meier survival of the promoter methylation of GSTK1.

We identified genetic alterations in GSTK1 in HNSC using the cBioPortal website. Of the 522 patients with HNSC, genomic alterations were found in nine patients, yielding an overall mutation rate of 1.7% (Figure 7A). Different types of GSTK1 genomic alterations did not resulting in changes in gene expression (Figure 7B). The above results suggest that genomic mutation was not the main mechanism for the alteration of GSTK1 gene expression. However, as shown in Figures 5D, E, the DNA methylation level of GSTK1 was high. GSTK1 expression was negatively related to hypermethylation level based on the cBioportal datasets (Spearman = −0.24, *p* < 0.001; Pearson = −0.28, *p* < 0.001; Figure 7C). Shallow deletion may be the major mechanism behind GSTK1 hypermethylation. MethSurv was used to evaluate the effect of hypermethylation levels on prognosis. We discovered that cg03879613, located on a CpG island, was associated with a poor prognosis (Figures 7D, E). Taken together, the above results suggest that GSTK1 methylation and differential expression do occur in HNSC tissue.

## Discussion

GSTK1, a member of the GST family, can detoxify xenobiotics, environmental carcinogens, and reactive oxygen species (Henderson et al., 1998; Morel and Aninat, 2011). The role of GSKT1 in tumors has been the subject of recent research. Prior studies found that GSTK1 is related to the tumorigenesis of breast, sarcoma, and prostate tumors (Mavis et al., 2009; Luthra et al., 2018; Quan et al., 2022). However, there are few studies on GSTK1 in HNSC. In light of this, we decided to perform a fully integrated bioinformatics analysis to identify the prognostic utility and potential therapeutic targets of GSTK1 in HNSC.

The first phase of our study used the TIMER and TCGA databases to compare the expression levels of GSTK1 in cancerous and normal tissues. GSTK1 expression in HNSC, BRCA, LUSC, and THCA tumors was significantly lower than in normal tissues, which was consistent with previous studies on sarcoma and breast cancer (Luthra et al., 2018; Quan et al., 2022). Quan et al. found that GSTK1 could scavenge reactive oxygen species through the antioxidant defense machinery, thereby reducing cancer risk (Quan et al., 2022). Longhitano et al. reported that increased GSTK1 can mediate lactate metabolism and oxidative stress *via* crosstalk between GPR81/IGFBP6, thereby inhibiting breast cancer progression (Longhitano et al., 2022). These previous findings suggest that GSTK1 functions as an anti-oncogene.

Patients with breast cancer and sarcomas with low GSTK1 expression had worse prognoses (Luthra et al., 2018; Quan et al., 2022). However, associations between GSTK1 expression and prognosis have not been well described in other cancer types. The present work compared the expression GSTK1 in normal tissue versus human cancers to determine if it is potential prognostic cancer biomarker. The Kaplan–Meier plotter and HPA databases indicated that, although survival results showed that low GSTK1 expression was significantly related to poor survival in BLCA, KIRP, and HNSC, only in patients with HNSC was the expression level of GSTK1 protein decreased in the tumor compared with the normal tissues around it. This indicates that GSTK1 might play a role in the growth and progression of HNSC. Multivariate Cox analysis further confirmed that low GSTK1 expression was an independent risk factor for shorter OS in patients with HNSC, suggesting the independent prognostic value of GSTK1 for HNSC. HNSC is a complex and heterogeneous tumor that is characterized by multiple genetic mutations, DNA damage repair, epigenetic alterations, and chromosomal deletions (Ang et al., 2010; Ramos et al., 2010). The primary clinical treatments for advanced HNSC are currently surgery, chemotherapy, and radiotherapy. Due to a lack of early diagnosis strategies and effective therapeutic targets, these approaches have a limited impact on survival. Most patients eventually die from cancer metastases and chemotherapy resistance (Pisani et al., 2020). The effect of GSTK1 on HNSC may therefore have both prognostic and therapeutic value.

We found that HNSC patients with T stage 3 or 4 had lower GSTK1 expression than those with T stage 1 or 2 disease. These results may indicate that low GSTK1 expression is suggestive of a high-grade tumor stage and can be used as a prognostic biomarker for HNSC. Our results also showed that GSTK1 expression level was lower in patients with a history of smoking or significant alcohol intake. Smokers and drinkers would impair the detoxifying system of GSTs, potentially increasing their susceptibility to carcinogenesis (Soares et al., 2017). Many previous studies have shown that tobacco and alcohol are related to HNSC carcinogenesis and reduce the efficacy of tumor-targeted therapy (Jethwa and Khariwala, 2017; da Silva Souto et al., 2021). Multivariate logistic regression further confirmed that T stage and smoker history were independent significant factors of GSTK1 expression. Thus, our findings suggest that tobacco and higher T stage patient groups with HNSC should be considered independently for targeted treatments.

To further investigate the role of GSTK1 in HNSC, we initially proved that the expression of GSTK1 in HNSC tumor specimens was lower than that of normal para-carcinomatous tissues. These results indicate that lower GSTK1 expression was significantly associated with HNSC tumorigenesis and progression. TCGA data were used for GSEA, which showed that GSTK1 expression was related to immune infiltration and DNA methylation pathways. GSTK1 expression was positively enriched in immune infiltration pathways, including primary immunodeficiency (PID), immunoregulatory interactions between lymphoid and non-lymphoid cells (IGI), and interferon gamma signaling (IFN-γ). PID refers to a large heterogeneous genetic code that results from defects in immune system development, (McCusker et al., 2018). PID patients often have a strong predisposition to cancer due to the genomic instability created by defective DNA repair mechanisms (de Miranda et al., 2011). The IGI pathway plays a key role in modifying the response of lymphoid cells to self and tumor antigens (Nedvetzki et al., 2007). The IGI pathway could fight against tumorigenesis and metastasis and was recognized as a potential target for immunotherapy in cancer patients (Shen et al., 2020). IFN-γ is also an antitumor cytokine that facilitates immunosurveillance against tumor cells (Bhat et al., 2018). IFN-γ-induced activation of JAK2-STAT1 results in an anti-proliferative response against tumor cells, and has implications in the design of targeted anti-cancer therapies (Gao et al., 2018). Two pathways related to DNA methylation were negatively correlated with GSTK1 expression: PRC2 methylates histones (PRC2) and DNA methylation. PRC2 catalyzes the methylation of histone H3 on lysine 27 to generate trimethyl-H3K27 marks, thereby leading to a repressive chromatin state that inhibits gene expression (Shi et al., 2019). Enhancer of zeste homolog 2 (EZH2) is an enzymatic catalytic subunit of PRC2 that is currently a hot research topic for cancer therapy (Duan et al., 2020). DNA methylation is important to imprinting, X-inactivation, cancer, and the developmental control of gene expression (Bergman and Cedar, 2013). Local hypermethylated alterations within gene promoters can result in protein expression changes that contribute to the development of different cancer phenotypes (Li et al., 2015). The above studies showed that GSTK1 may affect the progression of HNSC by regulating immune infiltration, and that DNA methylation may be the potential regulatory mechanism behind GSTK1 expression.

We further explored correlations between GSTK1 expression and immune cells infiltration in the setting of HNSC. GSEA analysis and IHC of clinical specimens showed that the high expression of GSTK1 was positively correlated with T cells and Cytotoxic cells infiltration. It is clear that most T cells and Cytotoxic cells in the network have a significant positive impact on cancer outcomes. A novel cytokine-induced cell killing therapy proposes using T cells preactivated by cytokines such as IFN-γ to recognize and kill tumor cells through innate immune receptors such as NKG2D (Meier et al., 2022). Cytotoxic cells, commonly known as CD8 T cells, are the preferred immune cells for targeting cancer. Upon activation, effector CD8 T cells infiltrate to the core or invading site of the tumor and play an essential role in killing cancer cells. In patients with HNSC, eliciting the maximum possible tumor-reactive CD8 T cells response could be considered a target of vaccine antigens to tumor immunotherapy (Eberhardt et al., 2021). The above findings indicate that GSTK1 plays an essential regulatory role in the tumor immune microenvironment and exerts an antitumor effect by recruiting immune cells, especially T cells and Cytotoxic cells.

The alteration of gene expression is involved in cancer development, and growing evidence suggests genetic mutation and epigenetic changes play critical roles in regulating gene expression (Zhang et al., 2021). Our results showed that the mutation rate of GSTK1 gene in HNSC was low, which indicates that genomic mutation was not the main mechanism for the alteration of GSTK1 gene expression. Further analysis revealed that the change in GSTK1 expression resulted from epigenetic changes in DNA methylation, which indicated that the role of GSTK1 on tumorigenesis was more closely related to DNA methylation. Aberrant DNA methylation has been observed in various cancers, resulting in the inactivation of certain tumor-suppressor genes within promoter regions or the silencing of a broad range of genes in different cancers (Klutstein et al., 2016). Studies have demonstrated that hypermethylation contributes to the tumorigenesis of various cancers, including gastric cancer, thyroid cancer, and breast cancer (Li et al., 2015; Zafon et al., 2019; Schabort et al., 2020). Our study showed that hypermethylation of GSTK1 is associated with low gene expression and poor HNSC prognosis. Mavis et al. showed that promoter DNA hypermethylation appeared to inhibit GSTK1 gene expression in the normal murine prostate, and that GSTK1 genes are extensively downregulated in primary transgenic prostate adenocarcinoma (Mavis et al., 2009). This indicates that DNA methylation could influence GSTK1 expression. DNA methylation is reversible, which makes it an interesting therapeutic target. We explored the methylation site of GSTK1, which is located in cg03879613 of CpG island. CpG island normally has a very low degree of methylation. Methylation of CpG island would affect the binding of proteins to the promoter region of GSTK1 mRNA, thereby limiting its expression (Meng et al., 2015). The development of anticancer epigenetic drugs targeting DNA methylation is an increasingly hot topic in cancer therapy research (Yang et al., 2021). Various therapeutic drugs have been developed for reversing methylation, opening an avenue toward curing cancers (Pan et al., 2018). For example, DNMTs inhibitors including azacytidine, decitabine, and zebularine are effective in the treatment of bone marrow disorders and lymphoid malignancies (Khan et al., 2013) and are considered to be radiosensitizers in cancers (Gnyszka et al., 2013; Zielske, 2015). Recently, Li et al. found that three compounds (LX-3, LX-4, and LX-5) could selectively activate the p38 mitogen-activated protein kinase (MAPK) pathway and active a subset of endogenous genes repressed by DNA methylation, which provides key compounds for the study of selective agonists of the p38 pathway and p38 MAPK–targeted genes repressed by DNA methylation (Li et al., 2018). Furthermore, Ye et al. proved *in vitro* and *in vivo* that DNA hypermethylation-induced miR-182 silencing targets BCL2 and HOXA9 to accelerate acute myeloid leukemia progression, thus providing a potential selective therapeutic target for acute myeloid leukemia patients (Ye et al., 2023). The present work provides evidence that GSTK1 hypermethylation and low levels of gene expression predict a poor HNSC prognosis. This can play an important role in guiding the development of novel epigenetic biomarkers, and in the identification of potential targets for future therapeutic interventions.

Although it presents comprehensive and systematic understanding of the relationship between GSTK1 and HNSC, our study had several limitations. First, our data was validated using the TCGA database. More external datasets are needed to validate our findings. Second, the methylation changes of GSTK1 in HNSC need to be detected by further experiments, such as conventional methylation-specific PCR, methylated DNA immunoprecipitation-sequencing, and so on. Third, further *in vivo* and cellular experiments are needed to confirm the effect of GSTK1 demethylation on the carcinogenesis of HNSC cells and HNSC therapy. Fourth, no DNA methylation therapeutic drug for GSTK1 has been evaluated clinically, which should be a focus of future research. We therefore plan to perform experiments to evaluate the benefits of drugs targeting GSTK1 methylation on cancer model survival and tumor growth inhibition.

## Conclusion

Downregulation of GSTK1 expression is closely related to poor HNSC prognosis. GSTK1 has certain reference values for the diagnosis and prognosis of HNSC. GSTK1 may affect the progression of HNSC by regulating immune infiltration. DNA methylation may be a potential regulatory mechanism of GSTK1 expression. GSTK1 may serve as a potential prognostic and therapeutic biomarker for HNSC.

## Data Availability

The datasets presented in this study can be found in online repositories. The names of the repository/repositories and accession number(s) can be found in the article/Supplementary Material.
