# Supplementary material for: Prognostic biomarker GSTK1 in head and neck squamous cell carcinoma and its correlation with immune infiltration and DNA methylation
Source: Front Genet. 2023 Mar 3;14:1041042. doi: 10.3389/fgene.2023.1041042 (PMC10020208; doi:10.3389/fgene.2023.1041042)
Supplement: Supplementary file 1 [file Table1.docx]

**Supplementary Material**

**Supplementary Table1** | Clinicopathological information of the HNSC patient sets in this study.

| **Baseline characteristics** | **Value*** |
| --- | --- |
| **Age (years)** | 54.6 (44-66) |
| Gender |  |
| Male | 8 (73) |
| Female | 2 (27) |
| **Smoking status** |  |
| Yes | 7 (64) |
| No | 4 (36) |
| **Alcohol history** |  |
| Yes | 7 (64) |
| No | 4 (73) |
| **Grade** |  |
| G1 | 2 (18) |
| G2 | 5 (45) |
| G3 | 3 (27) |
| G4 | 1 (10) |
| GX | 0 |
| **Stage** |  |
| I-II | 3 (27) |
| III-IV | 6 (55) |
| unknow | 2 (18) |

***values are expressed as median (range) or n (%)**
